# Fractional exhaled nitric oxide levels in relation to work‐related respiratory burden and sensitization to wheat flour and multigrain in bakers

**DOI:** 10.1002/clt2.12018

**Published:** 2021-10-06

**Authors:** Mario Olivieri, Mario Malerba, Gianluca Spiteri, Lorena Torroni, Carlo Alberto Biscardo, Dario Valenza, Andrei Malinovschi

**Affiliations:** ^1^ Unit of Occupational Medicine Department of Diagnostics and Public Health University of Verona Verona Italy; ^2^ Department of Translational Medicine University of Piemonte Orientale Novara Italy; ^3^ Unit of Epidemiology and Medical Statistics Department of Diagnostic and Public Health University of Verona Verona Italy; ^4^ Department of Medical Sciences Clinical Physiology Uppsala University Uppsala Sweden

**Keywords:** allergic sensitization, bakery, fractional exhaled nitric oxide, lower airway symptoms, occupational exposure, work‐related asthma, work‐related rhinitis

## Abstract

**Background:**

Work‐related lower airway symptoms (WR‐LAS), rhinitis (WRR), and asthma (WRA) are very common among bakers, due to airborne exposure to wheat flour and multigrain. Limited data is available regarding fractional exhaled nitric oxide (FeNO) in bakers in relation to respiratory burden and occupational sensitization in a real‐life situation.

**Objective:**

To analyze FeNO levels in relation to WRR, WR‐LAS, and WRA with regard to allergic sensitization to occupational allergen in bakers.

**Methods:**

Cross‐sectional, observational study of 174 bakers employed in traditional small bakeries in the Verona District. Subjects did FeNO measurements, spirometry, methacholine challenge, and skin prick test to common inhalant aeroallergens and bakeries occupational allergens.

**Results:**

FeNO levels were higher in subjects sensitized to occupational allergens compared with bakers not sensitized to occupational allergens (22.8 ppb (18.9, 27.6) vs. 12.0 ppb (9.9, 14.5), *p* < 0.05). FeNO levels were higher in bakers with WRR and occupational sensitization (25.4 (20.6, 31.3)) than in bakers with WRR without occupational sensitization compared and bakers without respiratory burden (13.4 (9.6, 18.6) and 11.9 (9.8, 14.5), both *p* < 0.001). Similar findings were found for WR‐LAS with regard to the same categories (31.2 (24.1, 40.4) vs 13.3 (11.4, 15.6) and 15.3 (8.5, 27.5), *p* < 0.001 and *p* = 0.005). Bakers with WRA, with or without occupational allergic sensitization, had higher levels of FeNO than bakers without respiratory burden (both *p* ≤ 0.001). These findings were consistent after adjustments for gender, age, height, weight, smoking, and sensitization to common inhalant aeroallergens and lung function.

**Conclusions:**

WRR and lower airway symptoms in bakers sensitized to occupational allergens relate to increased FeNO. Our study suggests that FeNO is associated with work‐related allergic inflammation in occupational sensitized bakers, but future studies are needed to assess how FeNO should be integrated in the diagnostic work‐up of occupational disease in bakers.

## 1 BACKGROUND

A high prevalence of work‐related allergic asthma in bakers is well known.^1^ Occupational rhinitis is even two to four times more common.^2^, ^3^ Moreover, both diseases frequently coexist.^4^ This relationship is due to airborne exposure in bakeries to wheat flour and consequent sensitization that results in onset of allergic symptoms at work.^5^ Small family‐run bakeries are generally characterized by poor automation, so production processes are often carried out manually (i.e., weighing, dumping, mixing bagged ingredients, and cleaning). To produce a large variety of bread, it is necessary to use many types of flour with additives or multigrain, a blend of several cereal flours, seed flours, and enzymes. Therefore, the probability of sensitizing and developing allergic respiratory symptoms when exposed both to flour and multigrain might increase with increasing exposure.^6^

Fractional exhaled nitric oxide (FeNO) is a marker of type 2 inflammation.^7^ FeNO reflects the production of nitric oxide (NO) in the respiratory epithelium by activation of inducible NO synthetase as response to different triggers, as for example, allergen exposure.^8^ FeNO is useful as an aid in diagnostic work‐up and the follow‐up of patients with asthma.^9^ FeNO appears to find a role in occupational asthma with recent studies suggesting use of FeNO both in relation to work exposures and standardized inhalation allergen challenges. An increase of 20 ppb in FeNO in relation to occupational exposure could be an additional diagnostic tool in the workup of asthma and help in establishing the diagnosis of occupational asthma in about 20% of the cases with suspected occupational asthma, according to a recent study.^10^ Increase of FeNO in relation to specific inhalation challenge with occupational allergens could be combined with lung function information and provided to be useful in the diagnostic workup of occupational asthma.^11^ A study among bakers, farmers and healthcare workers showed that a significant increase in FeNO occurred 24 h after a specific inhalation test.^12^ However, allergen inhalation challenges are performed to little extent, especially in bakers sensitized to several occupational allergens, and therefore it is of interest to study if FeNO measurements in real life reflect the degree of occupational sensitization and exposure.

Studies on the relationship between FeNO and respiratory symptoms/asthma in bakers are scant. In a population of apprentice bakers, an increase in FeNO is related to the onset of bronchial hyperresponsiveness (BHR) regardless of atopy, suggesting that the measurement of FeNO in workers exposed to agents is capable to identify workers at risk of occupational asthma.^13^ Few information is available on the prevalence and coexistence of work‐related nasal and asthma‐like disorders in bakers using enzymes and/or multigrain and their effects on FeNO. In supermarket bakers with allergic respiratory symptoms increased FeNO values were detected in sensitized to cereals and α‐amylase and wheat immunoglobulin E (IgE) levels accounted for most of the variability in FeNO.^14^ FeNO is well‐studied in relation to common inhalant aeroallergen sensitization, and higher levels have been reported especially with regard to allergic sensitization to perennial allergens.^15^ However, limited data is available regarding FeNO related to IgE sensitization towards occupational allergens due to many different factors that influence the levels of FeNO.

The aim of the present study was to analyze FeNO levels in bakers in relation to respiratory symptoms and sensitization to occupational allergens in bakeries.

## 2 METHODS

### 2.1 Population

A total of 229 traditional bakeries in Verona area were invited, through Bakers Labour Organization of Verona, to participate in a cross‐sectional questionnaire‐based survey finalized to a preventive medicine project. This resulted in 211 bakeries (92%) that accepted the invitation and 727 bakery employees who responded the questionnaire.^16^ All responding bakers were offered to participate in a clinical visit. The present study is a cross‐sectional, observational study based on 174 bakery workers that accepted to participate in the clinical visit. The study was approved by the local Ethics Committee (Prog. no. 1827).

### 2.2 Clinical visit

The clinical interview included a questionnaire based on the European Community Respiratory Health Survey.^17^ Smoking status was questionnaire‐assessed, and we classified the subjects as non‐ and current smokers. Additional questions concerning the onset of work‐related nasal and respiratory symptoms triggered by exposure to wheat flour and/or multigrain during the work shift and improved when away from work were included, according to literature.^18^, ^19^, ^20^, ^21^ The clinical visit was performed in the late morning on the same day that the subject has worked in the bakery.

### 2.3 Exhaled NO

FeNO was measured at 50 ml/s flow using a Chemiluminescence Analyzer (CLD88; Ecomedics). FeNO was measured in accordance with international guidelines, and hence before spirometry.^9^ The pulmonary technician performing both FeNO and pulmonary function testing was blinded to the answers from the questionnaire.

### 2.4 Pulmonary function testing

Each patient underwent baseline spirometry (SensorMedics V‐Max 22) in accordance with the ATS/ERS guidelines. In subjects without airway obstruction, methacholine challenge (MB3 Dosimeter; Mefar) was performed according to guidelines.^22^ The European Coal and Steel reference values have been used.^23^

The methacholine challenge was stopped when a cumulative dose of 2 mg of methacholine was reached or when the forced expiratory value at 1 s (FEV_1_) had fallen by 20% or more below the best baseline FEV_1_ following diluent inhalation (PD_20_ FEV_1_). The test was considered as positive for a provocative dose of methacholine (PD_20_ FEV_1_) ≤1 mg.^24^ We have used the definition proposed by ERS for lower limit of normal for FEV_1_/FVC (88% of predicted for men and 89% of predicted for women).^25^

### 2.5 Allergen sensitization

#### 2.5.1 Skin prick tests

Common inhalant aeroallergen skin prick test (SPT) extracts included pollen (birch, olive tree, grass, *Parietaria*, *Artemisia*, *Ambrosia*, cat dander, *Dermatophagoides pteronyssinus*) and *Dermatophagoides farinae*, *Alternaria alternata*, *Cladosporium herbarum* (Lofarma). Bakery allergens (yeast, wheat flour, rye flour, barley flour, oat flour, and soy flour) (Lofarma) and *α*‐amylase allergen (Stallergenes) were also tested. Histamine phosphate (10 mg/ml) and normal saline were used as positive and negative controls, respectively. Positive SPT was defined as a weal diameter ≥3 mm. Allergic sensitization to common inhalant aeroallergens was defined if at least one positive SPT was found to any of the common inhalant aeroallergens included.

#### 2.5.2 Case classification

The following respiratory symptoms: cough, wheezing, chest tightness, and shortness of breath as well as nasal symptoms (sneezy, runny, or blocked nose in absence of a cold) triggered by exposure to wheat flour and/or multigrain were assessed to be work related if worsening during the work shift and improving when away from work.

WRR: presence of work‐related nasal symptoms with or without sensitization to any occupational allergens.

WR‐LAS: presence of work‐related lower airway symptoms with or without sensitization to any occupational allergens.

WRA: presence of work‐related asthma‐like symptoms and airway obstruction and/or positivity to the nonspecific bronchoprovocation test (PD_20_ FEV_1_ < 1000 mcg) with or without sensitization to any occupational allergens. A total of five subjects could not be classified due to missing needed information.

### 2.6 Statistical analysis

Statistical analyses were performed using STATA 15.1 (Stata Corp.). A *p* < 0.05 was considered as statistically significant.

Unpaired *t*‐test of log‐transformed FeNO levels was used for comparisons between different groups. FeNO levels are presented as geometric mean (95% confidence interval).

Multiple logistic regression models were used to study the relation between work‐related rhinitis (WRR), work‐related lower airway symptoms (WR‐LAS), and work‐related asthma (WRA) with and without occupational sensitization in relation to FeNO after adjustments for gender, age, height, weight, smoking, and allergic sensitization to common inhalant aeroallergens. Further adjustment for FEV_1_/FVC was used in an additional model. Similar model was used for analyzing the relation of FeNO with presence of WRR and WRA and occupational sensitization (Table1). Furthermore, percentual increases in FeNO, with regard to presence of WRR, WRA and occupational sensitization and compared with the group without WRR and WRA, were defined^26^ and presented for simple and multiple linear regression models.

**TABLE 1:** FeNO levels and percentual FeNO increase^a^ in relation to WRR, WRA and occupational sensitization

	FeNO levels (geometric mean (95% CI))	Percentual increase compared with the group without WRR and WRA (univariate analyses)	Percentual increase compared with the group without WRR and WRA (multivariate analyses)^b^
Subjects without WRR and WRA (*n* = 56)	11.6 (9.6, 14.0)	–	–
WRR without occcupational sens (*n* = 29)	10.7 (7.5, 15.1)	−3% (−17%, 13%)	−2% (−16%, 14%)
WRR with occupational sens (*n* = 47)	17.5 (13.6, 22.5)	20% (5%, 37%)	17% (3%, 34%)
WRA ± WRR without occup sens (*n* = 6)	39.6 (21.2, 74.0)	71% (27%, 129%)	62% (22%, 116%)
Both WRR and WRA with occup sens (*n* = 31)	46.2 (34.8, 61.3)	82% (56%, 113%)	81% (55%,112%)

Abbreviations: FeNO, fractional exhaled nitric oxide; WRA, work‐related asthma; WRR, work‐related rhinitis.

^a^ Compared to subjects without WRR and WRA.

^b^ Adjusted for gender, age, height, weight, smoking and common inhalant aeroallergen sensitization.

## 3 RESULTS

A total of 174 bakery workers working in bakery for a median of 11 years (interquartile range: 5–21 years) have been included in the present study. The subjects' characteristics are given in Table 2.

**TABLE 2:** Population characteristics

Male, *N* (%)	125 (71.8%)
Age, mean ± *SD*	40.2 ± 11.3
Height, mean ± *SD*	171 ± 8.7
Weight, mean ± *SD*	76 ± 14.2
BMI, mean ± *SD*	26.1 ± 4.6
Current smoking, *N* (%)	43 (24.7%)
FEV_1_ (% predicted)	100.3 ± 15.6
FEV_1_/FVC (% predicted)	96.6 ± 9.8
FEV_1_/FVC < LLN	21 (12.1%)
Methacholine challenge (PD_20_ ≤ 1 mg)	40 (24.5%)
FeNO ppb, GM (95% CI)	17.3 (15.0, 19.9)
Common inhalant aeroallergen sensitization^a^	117 (67.2%)
Occupational sensitization^b^	99 (56.9%)
Work‐related nasal symptoms (WRR)	116 (66.7%)
Work‐related lower airway symptoms (WR‐LAS)^c^	67 (38.5%)
Work‐related asthma (WRA)^d^	37 (21.9%)

*Note*: The percentages are shown in brackets. Eleven subjects did not perform methacholine challenge.

Abbreviations: BMI, body mass index; CI, confidence interval; FeNO ppb, fractional exhaled nitric oxide (parts per billion); GM, geometric mean; PD_20_: cumulative dose causing a 20% fall in FEV_1_.

^a^ Sensitization to common inhalant aeroallergens.

^b^ Sensitization to any bakery allergens.

^c^ WR‐LAS include any of the following symptoms in the work‐place: wheeze, chest tightness, shortness of breath.

^d^ Five subjects could not be classified.

### 3.1 FeNO in relation to anthropometric variables

FeNO was not significantly related to either height, weight, or BMI. No difference in relation to gender was found: 18.1 ppb (15.3, 21.4) in males versus 15.3 ppb (11.7, 20.3) in females (*p* = 0.31.)

### 3.2 FeNO in relation to smoking habits

Subjects currently smoking had lower levels than nonsmoking subjects: 12.3 ppb (8.8, 17.2) versus 19.3 ppb (16.6, 22.5) respectively (*p* = 0.007). Number of daily smoked cigarettes did not significantly relate with levels of FeNO (*p* = 0.11).

### 3.3 FeNO in relation to lung function and BHR

A weak relation of increased FeNO with lower FEV_1_ (% predicted) was found in all subjects (*r* = −0.16, *p* = 0.04). Increased FeNO was also related with lower FEV_1_/FVC ratio (*r* = −0.31, *p* < 0.001) and subjects with low FEV_1_/FVC ratio had higher levels than subjects with normal FEV_1_/FVC ratio: 39.7 ppb (26.4, 59.6) versus 15.4 ppb (13.3, 17.8) (*p* < 0.001).

A significant negative association between FeNO and PD_20_ was found in all subjects (*r* = −0.50, *p* < 0.001). Subjects with BHR (PD_20_ < 1 mg) had higher FeNO levels than subjects without BHR: 37.6 ppb (28.7, 49.2) versus 12.7 ppb (11.0, 14.7) (*p* < 0.001).

### 3.4 FeNO in relation to sensitization to common inhalant aeroallergens

FeNO was increased in subjects sensitized to common inhalant aeroallergens compared with subjects not sensitized to common inhalant aeroallergens: 19.9 ppb (16.7, 23.8) versus 13.0 ppb (10.3, 16.3) (*p* = 0.005).

### 3.5 FeNO in relation to occupational sensitization

FeNO was elevated in subjects sensitized to all occupational allergens, compared with nonsensitized subjects, in univariate analyses, with exception for amylase (Table 3). Being sensitized to any occupational allergen was also related to higher levels of FeNO (Table 3) and being sensitized to only one occupational allergen or at least two occupational allergens resulted in increased levels of FeNO compared with subjects nonsensitized to occupational allergens: 18.1 ppb (13.0, 25.3) and 25.8 ppb (20.5, 32.4) versus 12.0 ppb (9.9, 14.5).

**TABLE 3:** FeNO levels (geometric mean (95% CI)) with regard to sensitization to respective occupational allergen (univariate analyses)

Type of allergen	Number (%) of positive tests	FeNO in sensitized subjects	FeNO in nonsensitized subjects	*p* Value
Wheat	81 (46.7%)	24.7 (20.1, 30.3)	12.7 (10.6, 16.0)	<0.001
Rye	35 (20.1%)	29.3 (21.5, 40.0)	15.1 (13.0, 17.7)	<0.001
Barley	41 (23.6%)	27.0 (20.0, 36.6)	15.1 (12.9, 17.6)	<0.001
Oat	32 (18.4%)	24.0 (17.0, 33.8)	16.1 (13.7, 18.8)	0.03
Amylase	36 (20.7%)	22.3 (16.2, 30.6)	16.2 (13.8, 19.0)	0.07
Yeast	14 (8.1%)	28.9 (13.9, 60.1)	16.5 (14.3, 19.1)	0.03
Any allergen	99 (56.9%)	22.8 (18.9, 27.6)	12.0 (9.9, 14.5)	<0.001

Abbreviations: CI, confidence interval; FeNO, fractional exhaled nitric oxide.

### 3.6 FeNO in relation to symptoms upon exposure

FeNO was higher in subjects presenting wheeze, chest tightness or shortness of breath upon exposure, compared with subjects without the respective symptom in univariate analyses (Table 4). Similarly, nasal symptoms upon exposure related with higher FeNO levels (Table 4).

**TABLE 4:** FeNO levels (geometric mean (95% CI)) with regard to respiratory symptoms upon exposure

Symptom	Number (%)	FeNO if symptom present	FeNO if symptom absent	*p* Value
Wheeze	41 (23.6%)	35.5 (25.9, 48.6)	13.8 (12.0, 16.0)	<0.001
Chest tightness	33 (19.0%)	27.7 (18.5, 41.7)	15.5 (13.4, 17.9)	0.001
Shortness of breath	49 (28.2%)	32.6 (24.9, 42.8)	13.5 (11.6, 15.6)	<0.001
Cough	43 (24.7%)	20.3 (14.0, 29.4)	16.4 (14.2, 19.0)	0.21
Nasal symptoms	116 (66.7%)	20.8 (17.3, 25.0)	11.9 (9.8, 14.5)	<0.001

Abbreviations: CI, confidence interval; FeNO, fractional exhaled nitric oxide.

Having at least two asthma symptoms (of wheeze, chest tightness, and shortness of breath) was related to larger increase in FeNO than having only one asthma symptom or none: 35.9 ppb (25.6, 50.2) versus 17.5 ppb (12.7, 24.0) and 13.3 ppb (11.4, 15.6), both *p* < 0.001.

### 3.7 FeNO in relation to airway symptoms and sensitization by occupational allergens

**FIGURE 1:**
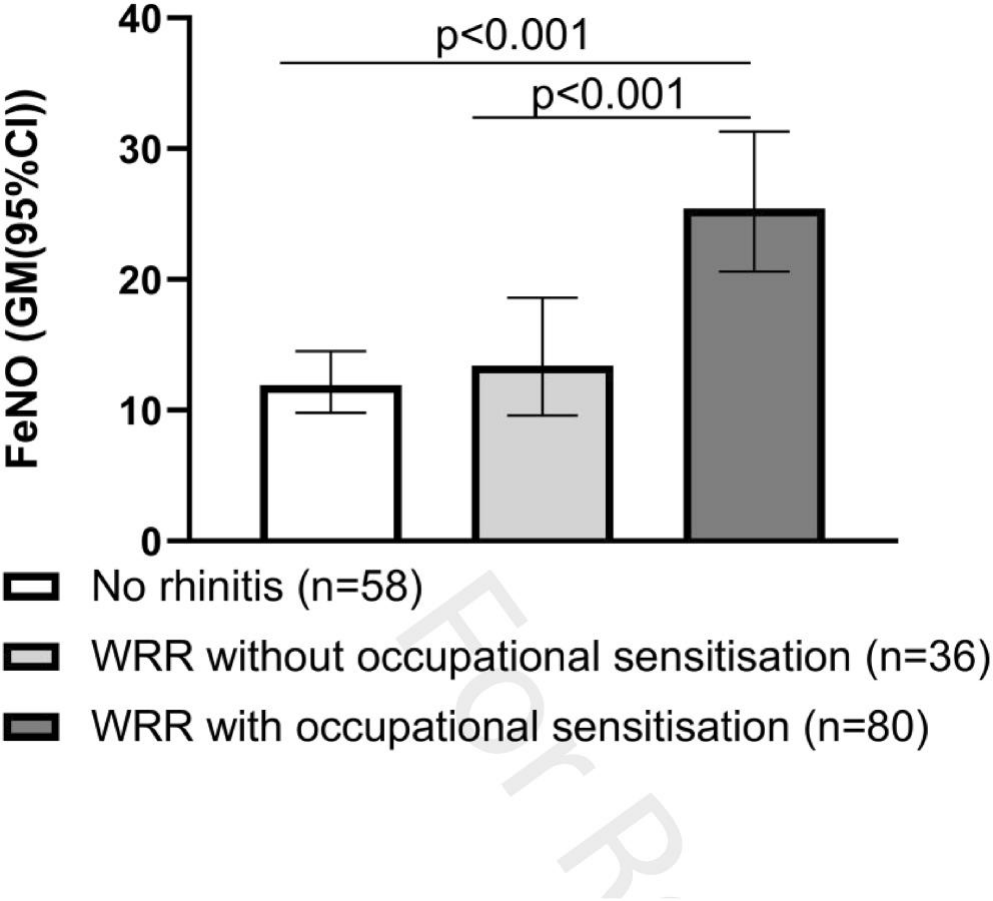
FeNO in relation to WRR and occupational sensitization. FeNO, fractional exhaled nitric oxide; WRR, work‐related rhinitis

FeNO was elevated only in subjects with WRR and sensitization to occupational allergens (*n* = 80), but not in subjects with WRR without occupational sensitization (*n* = 36), compared with subjects with no WRR (*n* = 58) (25.4 (20.6, 31.3) vs 13.4 (9.6, 18.6) and 11.9 (9.8, 14.5), both *p* < 0.001; Figure 1).

The difference between the group with WRR with occupational sensitization and the group without rhinitis, on one hand, and the group with WRR without occupational sensitization, on the other hand, were consistent for gender, age, height, weight, smoking, and common inhalant aeroallergen sensitization (*p* < 0.001 and *p* = 0.001, respectively). The results were consistent after further adjustment for FEV_1_/FVC (*p* < 0.001 and *p* = 0.006, respectively).

**FIGURE 2:**
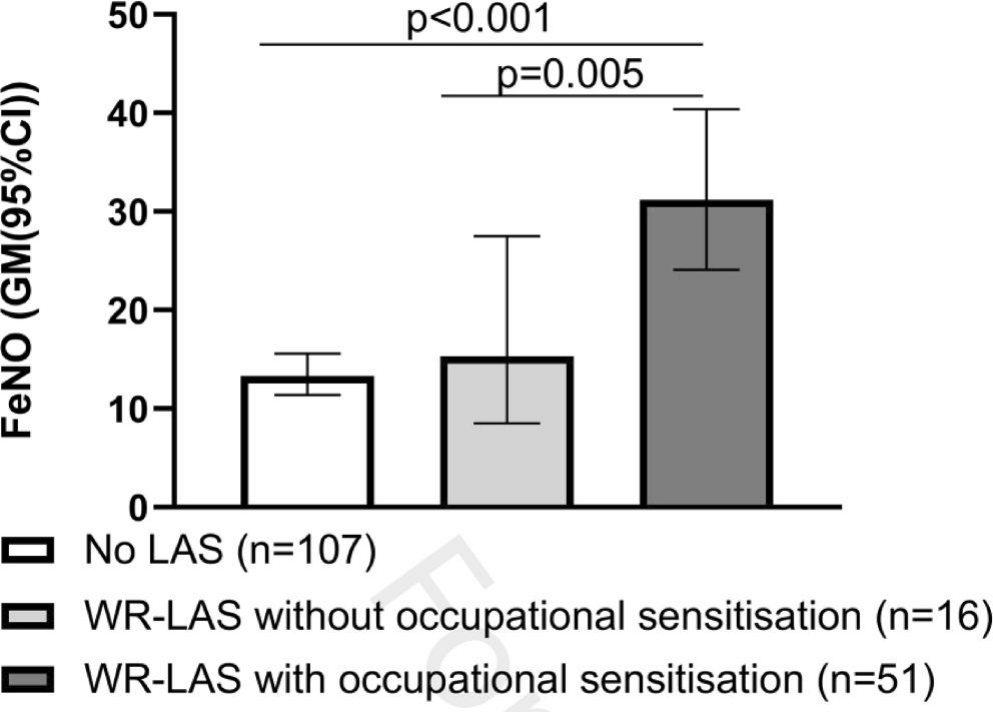
FeNO in relation to WR‐LAS and occupational sensitization. FeNO, fractional exhaled nitric oxide; work‐related lower airway symptoms

FeNO was elevated only in subjects with WR‐LAS (*n* = 51) that were sensitized to occupational allergens, but not in subjects without occupational sensitization (*n* = 16), compared with subjects without lower airway symptoms (*n* = 107) (Figure 2).

The difference between the group with WR‐LAS with occupational sensitization (*n* = 51) and the group without LAS (*n* = 107), on one hand, and the group with WR‐LAS without occupational sensitization (*n* = 16), on the other hand, were consistent for gender, age, height, weight, smoking, and common inhalant aeroallergen sensitization (31.2 ppb (24.1, 40.4) vs 13.3 ppb (11.4, 15.6) and 15.3 ppb (8.5, 27.5), *p* < 0.001 and *p* = 0.02, respectively). After further adjustment for FEV_1_/FVC, the difference between the group with WR‐LAS with occupational sensitization and the group without LAS was consistent (*p* < 0.001). The difference between the group with WR‐LAS without occupational sensitization and the group without LAS was not statistically significant (*p* = 0.07). No differences between WR‐LAS without occupational sensitization and subjects without LAS were found neither in univariate or multivariate analyses.

**FIGURE 3:**
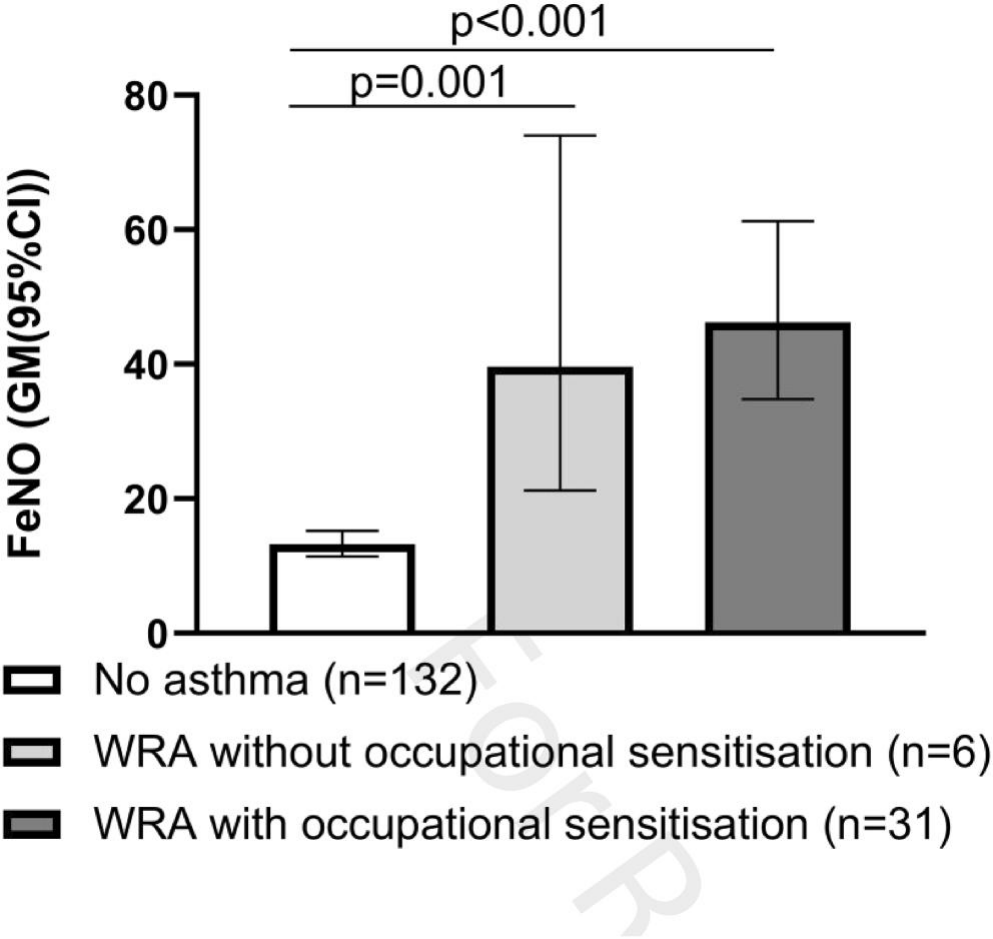
FeNO in relation to WRA and occupational sensitization. FeNO, fractional exhaled nitric oxide; WRA, work‐related asthma

FeNO was elevated both in subjects with WRA without (*n* = 6) and with sensitization to occupational allergens (*n* = 31), compared with subjects with no asthma (*n* = 132) (39.6 (21.2, 74.0) and 46.2 (34.8, 61.3) versus 13.2 (11.4, 15.2), both *p* ≤ 0.001) (Figure 3).

Both the group with WRA without occupational sensitization and the group with WRA with occupational sensitization had higher FeNO levels than the group without asthma after adjustments for gender, age, height, weight, smoking, and common inhalant aeroallergen sensitization (*p* = 0.003 and *p* < 0.001, respectively). The results were consistent after further adjustment for FEV_1_/FVC (*p* = 0.003 and *p* < 0.001, respectively).

### 3.8 FeNO in relation to years working in bakery

No relation between FeNO and years working in bakery was found (*p* = 0.48).

### 3.9 FeNO in relation to asthma, rhinitis, and sensitization to occupational allergens

Subjects with WRA, disregarding occupational allergic sensitization status had higher levels of FeNO than subjects without asthma and rhinitis (Table 1). The results from the univariate analyses are presented also as FeNO percentual increase, having as reference the group without WRR and WRA, in Table 1 These results were consistent after adjusting for potential confounders, such as gender, age, height, weight, smoking, and common inhalant aeroallergen sensitization (Table 1). The results were consistent for further adjustments for FEV_1_/FVC (data not shown).

## 4 DISCUSSION

FeNO levels were significantly higher in bakers sensitized to any occupational allergens than bakers not sensitized to bakery allergens. A novel finding was the dose–response effect on FeNO with larger increase in bakers sensitized to at least two occupational allergens compared to bakers sensitized to any bakery allergens. FeNO levels were significantly elevated in bakers with WRR and WR‐LAS only when they had allergic sensitization to occupational allergens. These relations were consistent after adjustments for allergic sensitization to common inhalant aeroallergens and smoking habits, two major determinants of FeNO levels. Higher FeNO were found in WRA in both subjects with and without allergic sensitization to occupational allergens. Finally, a novel finding of our study was the gradual increase of FeNO in subjects sensitized to occupational allergens with WRR without WRA that further increase in subjects sensitized to occupational allergens with both WRR and WRA.

Higher FeNO levels in supermarket bakery workers sensitized to wheat and rye flour has been reported by Baatjies et al.^14^ Our results confirm higher FeNO levels in bakers sensitized to any bakery allergen than not sensitized bakers. In line with this, we have detected increased FeNO values measured several hours after common daily flours exposure in bakery, accordingly to previous studies focusing on exposure to occupational allergens and demonstrating an increase of FeNO, measured 24 h after a single acute exposure.^27^ We could report a dose–response relation between FeNO and allergic sensitization to occupational allergens, in line with results regarding sensitization to multiple common inhalant aeroallergens.^15^ In line with thus, by using the “in vivo” SPT model, the association of two different allergens can increase the magnitude of the wheal skin response, in comparison with single allergen.^28^ Similarly IgE with different specificities have an additive effect on the release of mediators from basophils of polysensitized subjects.^29^

Bakers with WRR sensitized to occupational allergens had higher levels of FeNO compared to bakers with WRR without sensitization to occupational allergens or to bakers without WRR.^14^ This finding is consistent with the higher FeNO levels found in sensitized bakers with work‐related nasal symptoms and with the joint airway concept for inflammation in the lower and upper airways.^30^ Similar results are reported in our WR‐LAS bakers where the increase in FeNO levels is found only in bakers sensitized to professional allergens. However, the role of FeNO in occupational rhinitis is not well defined although FeNO measurement could be useful to diagnose the etiology of work‐related nasal and respiratory symptoms, more likely to be due to a nonspecific irritant effect of high total dust levels, rather than due to inflammatory allergy. Accordingly, several studies have confirmed that occupational awareness is present only in 15%–30% of bakers with work‐related symptoms.^21^, ^31^ The lack of a positive IgE reaction could be due to sensitization to until now unknown baking allergens.^32^

Subjects with WRA had increased FeNO levels and this was found both for subjects with and without sensitization to occupational allergens. Our results are in agreement with the recent view on FeNO as aid in diagnosis of occupational asthma^11^, ^33^ as FeNO could be proven both to increase after standardized inhalation challenges^11^ and that serial FeNO measurements during work and nonwork periods provide complementary information to diagnose occupational asthma in individual cases.^10^ That both WRA without and with occupational sensitization related to increased FeNO might be due to the fact that these subjects already developed airways obstruction or had BHR as this was part of our asthma definition. In line with this, we found significant inverse relationship among FeNO levels and airway obstruction, evaluated as a lower FEV_1_/FVC ratio or positive BHR, in agreement with literature.^34^ However, it has to be highlighted that the large majority of cases with asthma were seen in subjects sensitized to occupational allergens. The small size of the group with asthma without occupational sensitization warrants some caution in interpreting the results in that group.

Work‐related cough is not associated with increased levels of FeNO. This data agrees with literature concerning cough as a nonspecific symptoms of asthma. Normal levels of FeNO in patients with cough might also exclude a diagnosis of eosinophils bronchitis, in some rare cases due to occupational sensitization to wheat flour or enzymes in bakers referring cough in the absence of bronchial hyperreactivity, normal lung function, and eosinophils inflammation in the airways.^35^

The strength of our study is the detailed characterization of allergic sensitization with use of many common inhalant aeroallergens and occupational allergens present in bakeries. The design of the study was another strength of the study with performing of SPTs, FeNO and spirometry in 1 day during the clinical visit after the natural exposure in the bakery during the same morning. However, our study has also weaknesses. Due to the cross‐sectional design, only associations can be reported and no information can be provided regarding changes in exposure levels and FeNO increase/development of symptoms. Moreover, we cannot provide practical advice how FeNO should be integrated in the diagnostic work‐up of asthma and which FeNO values should be used at one time‐point assessment as FeNO is affected by both anthropometric characteristics, but more important smoking and sensitization to common inhalant. A Global Lung Function Initiative taskforce is currently working on reference values for FeNO and these might provide a good reference to express FeNO as *z*‐scores after accounting for these factors. Having FeNO measurements outside the occupational exposure period as a baseline FeNO for the individual might provide a possibility of assessing the work‐related FeNO increase. Furthermore, the natural exposure in the bakery to a mixture of different allergens does not allow for identification of the individual exposure effects on FeNO increase. Finally, a response bias towards a higher proportion of individuals with more occupational‐related symptoms participating in the clinical study is likely and expected. However, this is unlikely to have influenced our analyses that are focused on the relation between FeNO and work‐related symptoms/disease as the participants were unaware of their FeNO values after the questionnaire phase.

## 5 CONCLUSION

Our study confirms the association of FeNO with work‐related respiratory symptoms and disease in bakers sensitized and exposed to occupational allergens. Bakers sensitized to occupational allergens had increased FeNO levels if WRR was present and further increased levels of FeNO if work‐related asthma was present. Future studies need to focus on the diagnostic work‐up of WRR and asthma and which cut‐offs should be used in the assessment of occupational respiratory disease in bakers.

## CONFLICT OF INTERESTS

The authors declare that they have no conflict of interests.

## AUTHOR CONTRIBUTIONS

Mario Olivieri and Andrei Malinovschi conceived the present analyses. Mario Olivieri, Gianluca Spiteri, Carlo A. Biscardo, and Dario Valenza collected the data. Andrei Malinovschi analyzed the data. Mario Olivieri and Andrei Malinovschi wrote the first draft. All authors contributed with critical comments to the revision of the draft with regard to analysis and interpretation of the data. All authors read and approved the final manuscript.
